# Immunohistochemical and mRNA expression of RANK, RANKL, OPG, TLR2 and MyD88 during apical periodontitis progression in mice

**DOI:** 10.1590/1678-7757-2017-0512

**Published:** 2018-06-25

**Authors:** Driely BARREIROS, Carolina Maschietto PUCINELLI, Katharina Morant Holanda de OLIVEIRA, Francisco Wanderley Garcia PAULA-SILVA, Paulo NELSON FILHO, Lea Assed Bezerra da SILVA, Erika Calvano KÜCHLER, Raquel Assed Bezerra da SILVA

**Affiliations:** 1 Universidade de São Paulo, Faculdade de Odontologia de Ribeirão Preto, Departamento de Clínica Infantil, Ribeirão Preto, São Paulo, Brasil.; 2 Universidade Federal de Sergipe, Departamento de Odontologia de Lagarto, Lagarto, Sergipe, Brasil.

**Keywords:** RANK, RANKL protein, Osteoprotegerin, TLR2, MyD88 protein, Apical periodontitis, Mice

## Abstract

**Objective:**

To evaluate and correlate, in the same research, the mRNA expression and the staining of RANK, RANKL, OPG, TLR2 and MyD88 by immunohistochemistry in the apical periodontitis (AP) progression in mice.

**Material and Methods:**

AP was induced in the lower first molars of thirty-five C57BL/6 mice. They were assigned to four groups according to their euthanasia periods (G0, G7, G21 and G42). The jaws were removed and subjected to histotechnical processing, immunohistochemistry and real-time reverse transcription-PCR (qRT-PCR). Data were analyzed with parametric and nonparametric tests (α=0.05).

**Results:**

An increase of positive immunoreactivity for RANK, RANKL, OPG, TLR2 and MyD88 was observed over time (p<0.05). The RANKL expression was different between the groups G0 and G42, G21 and G42 (p=0.006), with G42 presenting the higher expression in both comparations. The OPG expression was statistically different between the groups G0 and G7, G7 and G21 and G7 and G42 (p<0.001), with G7 presenting higher expression in all the time points. The TLR2 expression was different between the groups G0 and G42 (p=0.03), with G42 showing the higher expression. The MyD88 expression presented a statistical significant difference between groups G7, G21 and G42 compared with G0 (p=0.01), with G0 presenting the smallest expression in all the comparisons. The Tnfrsf11/Tnfrsf11b (RANKL/OPG) ratio increased with the AP progression (p=0.002). A moderate positive correlation between MyD88 and RANKL (r=0.42; p=0.03) and between MyD88 and TLR2 (r=0.48; p<0.0001) was observed.

**Conclusion:**

The expression of the RANK, RANKL, OPG, MyD88 and TLR2 proteins as well as the ratio Tnfrsf11/Tnfrsf11b (RANKL/OPG) increased with AP progression. There was also a moderate positive correlation between the expression Myd88-Tnfrsf11 and Tlr2-Myd88, suggesting the relevance of Tlr2-Myd88 in bone loss due to bacterial infection.

## Introduction

Apical periodontitis (AP) represents an immunoinflammatory response of the host against root canal infection, which results in the destruction of the periodontal ligament, cementum, and alveolar bone surrounding the root apex^13^. In AP, the inflammatory process results in periapical bone resorption because of osteoclast activity^25^.

The receptor activator of nuclear factor kappa B (NF-κB), ligand (RANKL), its receptor, RANK, and osteoprotegerin (OPG) play crucial roles in regulating the differentiation, activation and survival of osteoclasts in physiological and pathological processes^17^. RANKL induces bone destruction, and its natural decoy receptor, OPG, protects against bone destruction by preventing the RANKL binding to its receptor RANK^9^.

Bone destruction in the AP progression occurs because of the host’s defense reaction, which is primarily caused by bacterial infection through root canals. The host’s defense reaction in AP is composed of several inflammatory cells, such as neutrophils, macrophages, and lymphocytes^29^, which synthesize biochemical mediators.

Toll-like receptors (TLRs) are type I transmembrane receptors that are strongly expressed in multiple cell types associated with infections of endodontic origin, such as neutrophils^23^, monocytes/macrophages, granulocytes, pulp fibroblasts, osteoclasts precursors, and mesenchymal cells^12^. TLRs exert an important role in the recognition of specific pathogen-derived components^30^ and transmit appropriate signals to the cells of the immune system^20^. The recognition of microorganisms, their components, and byproducts by TLRs stimulates the production of proinflammatory cytokines and costimulatory molecules, which are responsible for the different responses elicited by the identification of pathogen-associated molecular patterns^30^. TLRs are the initial step of the cascade activation in the recruitment of different adapter molecules^6^. One of these adapter molecules is the myeloid differentiation factor 88 (MyD88). The role of MyD88 as a universal adapter was proven by the induction of cytokine production upon stimulation with various ligands that activate different transmembrane receptors, all of which have the requirement for MyD88 recruitment in common^3^.

Several proinflammatory cytokines are associated with the regulation of osteoclast formation, osteoclastogenesis, and bone resorption^12^. The activation of TLRs by microbial ligands triggers specific intracellular signaling pathways that lead to increased release of inflammatory mediators, including those for the expression of RANK/RANKL/OPG proteins, the central mediators for the homeostasis of mineralized tissues^1^. Also, MyD88 is involved in the regulation of RANKL and OPG expression induced via TLR2 and TLR4 signaling^16^.

Although some studies had already evaluated these molecules, none of them correlate their gene expression and immunolocalization. For this reason, this study aimed to evaluate and correlate the gene expression and the immunolocalization of RANK, RANKL, and OPG.

## Material and methods

### Animals

This study was based on the ARRIVE guidelines for the reporting of animal studies^15^. All the animal procedures were performed according to the applicable ethical guidelines and regulations of the Animal Use Ethics Committee of the University of São Paulo - Campus of Ribeirão Preto (2014.1.911.58). Thirty-five male C57BL/6 mice, 6- to 8-weeks-old and 20 g in weight were used. Mice were obtained from the animal’s facility at the University of São Paulo, Campus of Ribeirão Preto, Brazil and were kept at the animal’s facility at the FORP/USP with free access to food and water.

### Apical periodontitis induction

AP was induced in both mandibular first molars (left and right) of the mice. The animals were anesthetized intramuscularly with ketamine 10% (150 mg/kg body weight - Agener União Química Farmacêutica Nacional S/A, Embu-Guaçu, SP, Brazil) and xylazine 2% (7.5 mg/kg, Dopaser, Laboratorios Calier SA, Barcelona, Catalonia, Spain). Then, the animals were placed in a specific surgical table for adequate visualization and easy access to the lower molars.

The induction of AP was based on a previously published protocol^27^. Both mandibular first molars were exposed to the oral cavity with stainless steel 1/4 round burs (GDK Densell Dental Technology, Buenos Aires, Argentina) in a low-speed handpiece to access the pulp chamber. After that, #8-K endodontic files (Maillefer S/A, Ballaigues, Jura-Nord Vaudois, Switzerland) were used to localize the root canals. The root canals were exposed to allow contamination by the oral microbiota.

**Figure 1:**
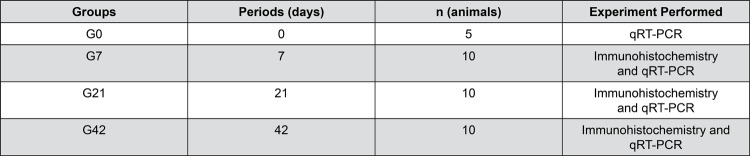
Description of animal groups

The mice were euthanized through intramuscular anesthesia with ketamine and xylazine and inhalation of CO_2_ in a specific chamber after the experimental periods of 0, 7, 21 or 42 d (Figure 1).

Then, the jaws were removed with sterile surgical scissors, obtaining two hemiarcades. The right one was submitted to histotechnical processing, while the left one was immersed in RNAlater solution^®^ (Ambion^TM^, Carlsbad, CA, USA) for the evaluation of gene expression through qRT-PCR technique.

### Histotechnical processing

The right lower jaws were removed and fixed in 10% phosphate-buffered formalin for 24 h at room temperature. Then, the pieces were washed in running water for 4 h and demineralized at room temperature in 4.13% ethylenediaminetetraacetic acid (pH=7.2), which was changed every week for 30 d. Once demineralized, the specimens were washed in running water for 2 h, dehydrated in ascending ethanol dilutions, cleared in xylol, and embedded in paraffin. Longitudinal 5-μm-thick semi-serial sections were cut in a mesiodistal orientation throughout the AP.

### Immunohistochemistry

The immunohistochemistry was performed according to previously published protocols^5^ ^,^ ^27^. The slides were incubated overnight with the primary antibodies (Santa Cruz Biotechnology Inc., Santa Cruz, CA, USA) diluted in 1% BSA: anti-RANK (polyclonal rabbit antibody H300 sc:9072, diluted 1:100; Santa Cruz Biotechnology Inc., Santa Cruz, CA, USA), anti-RANKL (polyclonal goat antibody sc:7628; Santa Cruz Biotechnology Inc., Santa Cruz, CA, USA; diluted 1:100), anti-OPG (polyclonal goat antibody n-20 sc:8468; Santa Cruz Biotechnology Inc., Santa Cruz, CA, USA; diluted 1:100), anti-TLR2 (polyclonal rabbit Bioss Antibod bs-1019R Santa Cruz Biotechnology Inc., Santa Cruz, CA, USA; diluted 1:100) and anti-MyD88 (polyclonal rabbit HFL-296 Santa Cruz Biotechnology Inc.; diluted 1:50). After returning to room temperature and being washed, the slides were incubated with a biotinylated secondary antibody (goat anti-rabbit IgG-B sc-2040 and rabbit anti-goat IgG-B sc-2774; Santa Cruz Biotechnology Inc., diluted 1:200) for 1 h at room temperature. The streptavidin-biotin-peroxidase complex (ABC kit, Vectastain; Vector Laboratories Inc., Burlingame, CA, USA) was then added for 30 minutes, followed by chromogen 3,3’ diaminobenzidine tetrahydrochloride hydrate (DAB; Sigma-Aldrich Corp., St. Louis, MO, USA), added with 3% hydrogen peroxide in PBS for 1 minute. The slides were counterstained with Harris’ hematoxylin.

The analysis was performed in Axio Imager.M1 microscope (Carl Zeiss MicroImaging GmbH, Göttingen, Lower Saxony, Germany) with 400X magnification under conventional light. The results were expressed in a qualitative manner, considering the presence/absence of immunostaining throughout the periapical lesion extension. Presence means positivity staining, thus considered when the cells exhibited brown coloration in the nucleus or in the cytoplasm. RANK, RANKL and OPG were observed in mono- and multinucleated cells and cementoclasts in the resorption gaps. MyD88 and TLR2 were observed in inflammatory cells, such as macrophages.

### qRT-PCR

**Figure 2:**
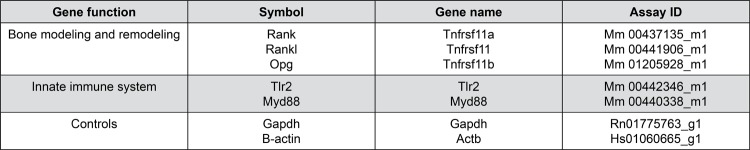
Description of studied genes

The left jaws were submitted to the extraction of nucleic acids (total RNA extraction) using a specific kit (PureLink RNA Mini Kit, Ambion, Life Technologies, Carlsbad, CA, USA). The procedures were performed according to the manufacturer’s protocol. The extracted RNA content was estimated in a spectrophotometer (Thermo Fisher Scientific Inc., Wilmington, DE) at a wavelength of 260 nm. Subsequently, RNA was converted into cDNA using a commercial kit (High Capacity cDNA Reverse Transcription Kit, Applied Biosystems, Lithuania). Briefly, messenger RNAs of RANK, RANKL, OPG, TLR2 and MyD88 were evaluated (Figure 2). GAPDH and β-Actin were used as reference genes. qRT-PCR reactions were performed in duplicate using TaqMan Gene Expression Assay (Applied Biosystems, Foster City, CA, USA). Relative quantification was performed using the 2^-ΔΔCt^ method.

### Statistical analysis

For immunohistochemistry, chi-square test was used to analyze the presence and absence according to the groups. The data for the expression of the genes were evaluated by group using the one-way ANOVA test, followed by the Tukey post-test. The RANKL/OPG ratio was also calculated only for mRNA expression.

The Spearman’s coefficient test was used to evaluate the degree of correlation between the genes expression. The strength of the positive and negative correlations was defined according to the value of the “Correlation Coefficient” (r) (1: perfect; 0.7 to 0.9: strong; 0.4 to 0.6: moderate; 0.1 to 0.3: weak; 0: no correlation).

All the analyses were performed using Graph Pad Prism 5.0 (Graph Pad Software Inc., San Diego, CA, USA). A significance level of 5% was set for all the analyses.

## Results

### Immunohistochemistry results

**Figure 3:**
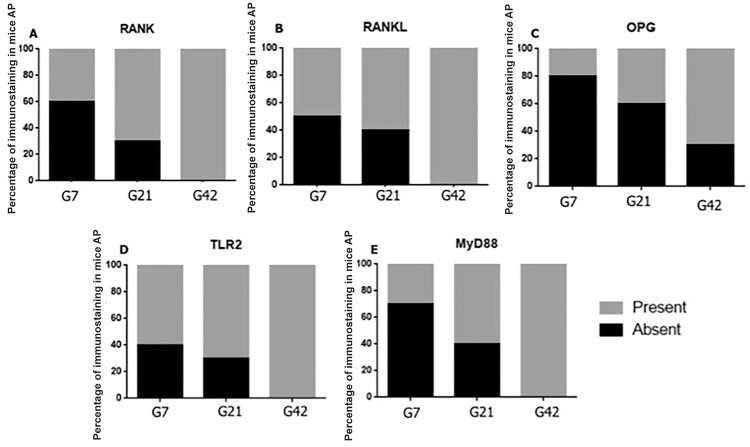
Graphical representation of immunostaining distribution (presence or absence) RANK (A), RANKL (B), OPG (C), TLR2 (D) and MyD88 (E) in the wild-type mice after periapical lesion induction in the periods of 7, 21 and 42 d

**Figure 4:**
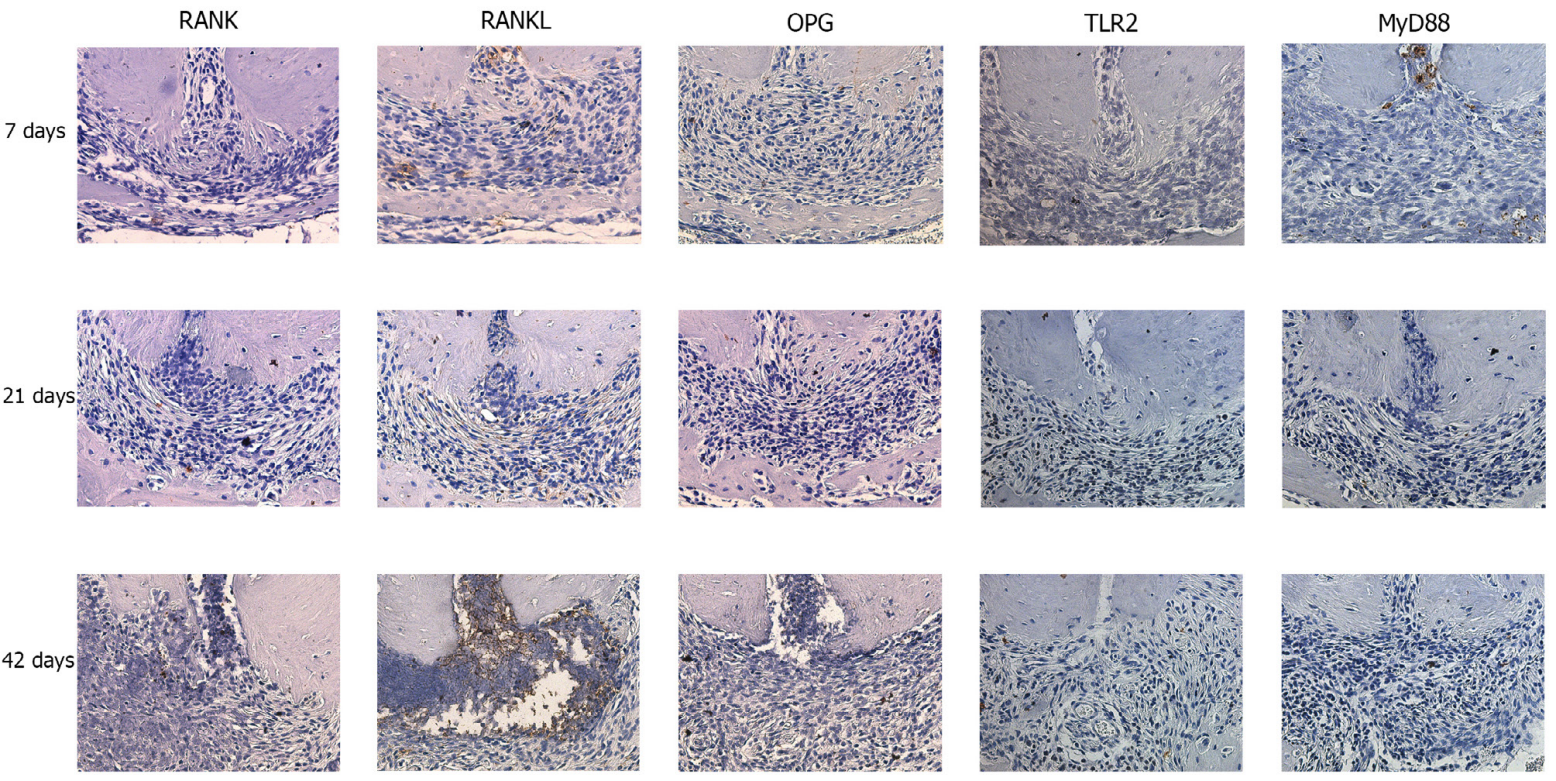
Representative photomicrographs obtained at 7, 21 and 42 d after the experimental induction of periapical lesions in wild-type mice. The presence or absence of immunostaining was determined by immunohistochemistry using RANK, RANKL, OPG, TLR2 and MyD88 (40x)

Figure 3 demonstrates the presence *versus* absence of the positive immunoreactivity for RANK (A), RANKL (B), OPG (C), TLR2 (D) and MyD88 (E) in G7, G21 and G42. Positive immunoreactivity increased for RANK, RANKL, OPG, TLR2 and MyD88 (p<0.05) during AP progression (Figure 4).

### Gene expression results

**Figure 5:**
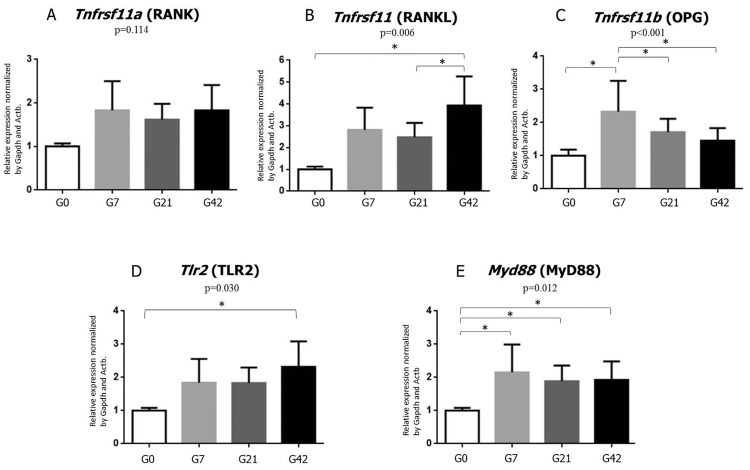
Graphical representation of mRNA expression distribution. Tnfrsf11a (A), Tnfrsf11 (B), Tnfrsf11b (C), Tlr2 (D) and MyD88 (E) in mice after AP induction in the periods of 0, 7, 21 and 42 d. The asterisk demonstrates statistical difference (p<0.05)

The RANK expression did not change over time (p=0.114) (Figure 5A).

The RANKL expression was different between the groups G0 and G42, G21 and G42 (p=0.006), with G42 presenting the higher expression in both comparisons (Figure 5B).

The OPG expression was different between the groups G0 and G7, G7 and G21 and G7 and G42 (p<0.001), with G7 presenting higher expression in all the time points (Figure 5C).

The TLR2 expression was different between the groups G0 and G42 (p=0.03), with G42 showing the higher expression (Figure 5D).

The MyD88 expression presented significant difference between groups G7, G21 and G42 compared with G0 (p=0.01), with G0 showing smaller expression in all the comparisons (Figure 5E).

### RANKL/OPG ratio

**Figure 6:**
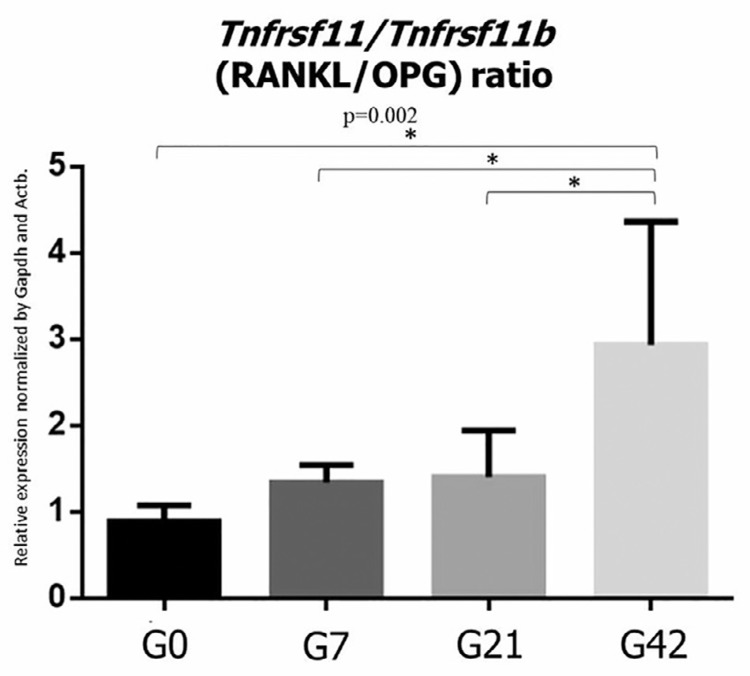
Graphical representation of Tnfrsf11:Tnfrsf11b ratio during AP progression. The asterisk demonstrates statistical difference (p<0.05)

Figure 6 shows the RANKL/OPG ratio according to the periods. The ratio of RANKL to OPG increased with the AP progression (p=0.002).

### Comparison between the results of immunohistochemistry and mRNA expression

After analysis of immunohistochemistry, RANK, RANKL and OPG increased their expression with a progression of the periapical lesion. Also, when mRNA results were analyzed, RANK expression was not statistically significant, but a RANKL/OPG ratio increased with periapical lesion progression. In addition, TLR2 expression increased in the immunohistochemistry and mRNA expression analysis. The same pattern was observed with MyD88 expression, which increased the expression along the progression of periapical lesion.

### Correlation results

There was a moderate positive correlation between MyD88 and RANKL (r=0.42; p=0.03). Also, a moderate positive correlation between MyD88 and TLR2 (r=0.48; p<0.0001) was observed (Table 1).

**Table 1:** Correlation between the mRNA expression of the genes

	Tlr2	Myd88
Tnfrsf11a	r=0.12 p=0.53	r=0.14 p=0.47
Tnfrsf11	r=0.27 p=0.17	r=0.42 p=0.03*
Tnfrsf11b	r=-0.09 p=0.64	r=0.22 p=0.26
Myd88	r=0.51 p<0.0001*	-

*bold indicates statistical difference (p<0.05)

## Discussion

In the last two decades, an increase in published articles regarding bone biology occurred. A noteworthy event was the identification and characterization of the RANK/RANKL/OPG system^14^ and its role in bone metabolism. Evidence of the importance of these molecules in the endodontic field has been growing since then^4^ ^,^ ^5^ ^,^ ^21^ ^,^ ^27^. Another important advance in endodontic research was the identification of receptors and adaptor molecules involved in innate immunity during AP progression^5^ ^,^ ^27^. TLR2 and MyD88 have an important role in pathogen recognition and activation of innate immunity^3^ ^,^ ^20^ ^,^ ^30^.

Published article^27^ characterized the formation and progression of experimentally induced AP lesions in TLR2-deficient (TLR2-/-)*.* They concluded that these animals developed larger periapical lesions with a greater number of osteoclasts compared with healthy animals, indicating the role of this receptor in the host’s immune and inflammatory response to root canal and periradicular infection^27^. Bezerra da Silva, et al.^5^ (2014), using MyD88-deficient (MyD88-/-) mice, analyzed the AP progression during 7, 21 and 42 d. They concluded that MyD88-/- mice developed larger periapical lesions when compared with wild-type mice. In addition, a higher number of osteoclasts in the AP were observed, and the RANK/RANKL/OPG system, through immunohistochemistry, was detected in all the experimental periods^5^.

In our study, the RANKL/OPG ratio increased according to the AP progression. The balance between RANKL and OPG expression is essential to determine the overall biological response in periodontal disease^28^, orthodontic tooth movement^18^, some osteolytic lesions in the facial skeleton^32^, and AP^4^ ^,^ ^21^. Menezes, et al.^21^ (2008) suggested that sites with active bone resorption show a differential pattern of RANKL/OPG expression when compared with sites where bone resorption is absent or minimal. Comparing these patterns of RANKL/OPG expression with those seen in periapical lesions, they could suggest that samples in which expression of RANKL predominates are putative progressive lesions, whereas samples in which expression of RANKL and OPG is similar or OPG is prevalent suggest groups whose lesions are potentially stable. At a certain time point during lesion development, the equilibrium of RANKL and OPG and the reduction of the osteoclast activation tend to occur, further resulting in a stable lesion.

Our results demonstrated that TLR2 presented the highest expression at 42 d of AP progression. In the literature, studies indicated that TLR2 is crucial for inflammatory bone loss in response to bacterial infection. For example, in chronic inflammatory periodontal disease, the *Porpyromonas gingivalis* is the primary organism associated with bone loss^34^. TLR2 also participates in signal transduction activated by the bacterial LPS of some pathogens such as *Porphyromonas gingivalis* ^35^ and *Porphyromonas endodontalis* ^31^ through binding with the MD-2 auxiliary molecule that is expressed in blood leukocytes and in different inflammatory cells participating in the immune response^8^. Apical periodontitis is, most frequently results.

Periapical periodontitis is the result of polymicrobial infection of pulpal origin, which leads to an inflammatory response within the periapical tissues and bone resorption lesion^19^, and some studies that evaluated the AP progression demonstrated that the animals developed extensive AP at 42 d^5^ ^,^ ^27^. Therefore, the high TLR2 expression at 42 d could be explained due to the presence of bacteria, which were involved in the activation of TLR2 signal transduction. Furthermore, *in vitro*, Ukai, et al.^34^ (2008) demonstrated TLR2 signaling in macrophage-induced osteoclastogenesis following live bacterial stimulation, and Silva, et al.^27^ (2012) demonstrated that TLR2-/- mice presented larger AP and higher number of osteoclasts. This suggested that TLR2 interacts with RANK/RANKL/OPG system during AP progression.

A previous study^27^ demonstrated that TLR2-/- mice developed larger periapical lesions with a greater number of osteoclasts compared with the WT animals. In parallel, TLR2 expression on macrophages was observed^33^. On the other hand, the TLR4 signaling depends on other co-receptors such as CD14, LBP, and MD2^2^ ^,^ ^10^ ^,^ ^22^. According to Rider, et al.^24^ (2016), TLR2 signaling is an endogenous suppressor of cellular CD14 expression. This mechanism prevents overactive CD14/TLR4-mediated inflammation against microbial infection. On the other side, Ukai, et al.^34^ (2008) concluded that *P. gingivalis* could induce the TLR2 expression on the cell surface, which results in the sensitization of macrophages. The ability of *P. gingivalis* to induce TLR2 cell surface expression may be a mechanism that contributes to the chronic inflammatory state induced by this pathogen.

Besides that, we found in this study that TLR2 expression was correlated with RANK, RANKL and OPG, in a moderate positive correlation between RANKL and MyD88. MyD88-mediated signal is essential for the osteoclastogenesis and is physiologically involved in bone turnover. MyD88-mediated signals induced RANKL expression in osteoblasts and supported the survival of osteoclasts induced by LPS^26^. Bone histomorphometry revealed that MyD88-deficient (MyD88-/-) mice exhibited typical osteopenia with reduced bone resorption and formation^26^. Based on the immunohistochemical analysis, similar levels of RANK, RANKL and OPG staining were observed in WT and MyD88-/- mice at 7, 21 and 42 d^5^.

MyD88-mediated signal is induced by TLR ligands^26^. Our results demonstrated a moderate correlation between TLR2 and MyD88. Previous studies showed that the overexpression of MyD88 causes a potent activation of RANKL, and the absence of MyD88 reduces RANKL activation^7^.

A recent study demonstrated that MyD88 is involved in the inhibition of OPG expression induced by LPS^16^. As has been demonstrated, LPS-stimulated osteoblasts induce RANKL expression through MyD88 activation^36^. Inflammatory cytokine- and LPS-induced expressions of RANKL are extremely dependent on MyD88^26^.

Briefly, inflammation of pulpal and periapical lesions is largely attributed to host’s inflammatory responses caused by bacterial infection of the pulp cavity and root canals^13^. Inflammatory response to bacterial infections causes increased osteoclastogenesis and bone loss mediated by TLRs^11^. Our study corroborates with this since we found that TLR2, as well as RANKL, increased with AP progression.

## Conclusion

The expression of RANK, RANKL, OPG, MyD88 and TRL2 was increased with periapical lesion progression. The mRNA analysis showed an increase in the ratio Tnfrsf11/Tnfrsf11b (RANKL/OPG) over time and a positive correlation between the expression of MyD88-Tnfrsf11 (MyD88-RANKL) and Tlr2-MyD88 (TLR2-MyD88). Although this study does not fully evaluate the molecular mechanisms of bone resorption during the periapical lesion, we hypothesize that these results demonstrate the relevance of TLR2 and MyD88 in bone loss from bacterial infection.
